# A Concise Clinical Application Strategy for Hyaluronic Acid Fillers in Facial Contouring and Rejuvenation

**DOI:** 10.1111/jocd.71011

**Published:** 2026-06-30

**Authors:** Hao Feng, Ting Wang, Caijin Xiang, Xiang Liu, Haihua Li, Yong Liao, Liangsen Zhao, Donghui Wu

**Affiliations:** ^1^ People's Hospital of Hunan Province (The First Affiliated Hospital of Hunan Normal University) Changsha China; ^2^ Kaifu Tongyan Aibo Medical Aesthetics Clinic Changsha China; ^3^ Changsha Yestar Medical Aesthetic Hospital Changsha China; ^4^ Shangrao Shangmei Medical Aesthetics Clinic Shangrao China; ^5^ Department of Medicine Bloomage Biotechnology Corporation Limited Beijing China; ^6^ Hunan Changsha Jiexi Medical Aesthetic Clinic Changsha China

**Keywords:** clinical application, facial rejuvenation, gel HA, granular HA, hyaluronic acid fillers, physicochemical properties, strategy

## 1 Introduction

Hyaluronic acid (HA) is the most widely used filler in aesthetic practice, with hundreds of brands and an even greater variety of formulations available worldwide. In China alone, more than 60 HA products have been officially approved for clinical use. The abundance of products inevitably presents both physicians and patients with complex choices. At times, selections are guided by brand reputation, as patients may request specific products recommended by friends or social media influencers. Price is another key factor. 1 mL syringes vary over twenty times in cost, so selections should fit patients' budgets. Physicians may also rely on habit and inventory, favoring products they use frequently and know well.

However, beyond brand and cost, the fundamental consideration in selecting HA fillers should be the ability to achieve optimal contour restoration and volumization. From this perspective, clinical indications for HA injection should be rationally categorized, with product selection guided by physicochemical properties appropriate to each indication. Drawing on personal clinical experience, the author classifies HA filler indications into five categories: contouring, volumization, smoothening, lifting, and revitalization. Contouring indications aim to accentuate osseous curves such as the supraorbital ridge, nasal dorsum, and mandibular line; volumization indications address soft tissue augmentation of areas like the temples and maxillary; smoothening indications include transitions in the forehead and subzygomatic hollow, where subtle smoothening rather than concentrated deposition is required; lifting indications involve lateral–superior injections to reposition inferomedial soft tissues; and revitalization indications refer specifically to dermal and superficial subdermal microinjections, focusing on fine replenishment and skin quality improvement.

The physicochemical properties of HA fillers encompass degree of crosslinking, elastic modulus (*G*′), viscous modulus (*G*″), cohesivity, swelling ratio, and particle size [1, 2]. In clinical practice, it is unrealistic for physicians to recall detailed parameters for every product, underscoring the need for a simplified classification to facilitate indication‐based selection. Classical studies have generally endorsed a two‐category system according to crosslinking technology and morphology: gel‐type (monophasic) and granular‐type (biphasic) fillers [3, 4, 5, 6]. These two groups display distinct macroscopic appearances, rheological behavior, and distribution patterns in tissue. In brief, granular fillers have higher *G*′ and lower cohesivity, tending to localize vertically in tissue, making them suitable for support and contouring. Gel fillers exhibit lower *G*′ and higher cohesivity, distributing more horizontally and thus better suited for volumization.

Granular HA fillers can be further subdivided by particle size into large, medium, and small [7]. Large particles measure approximately 600–1000 μm, small particles 300–500 μm, with medium particles falling in between. More recently, ultra‐small granular fillers have been introduced, with particle sizes ranging from 20 to 400 μm, smaller than traditional small particles. Particle size strongly influences in‐tissue support and the requirements for overlying tissue coverage and tightness: larger particles are more appropriate for deep placement and structural contouring, while smaller particles are suited for superficial or fine contour refinement. Improper placement—such as large particles in the superficial plane—may cause palpable nodules, whereas small particles in deep planes provide insufficient support.

**FIGURE 1:**
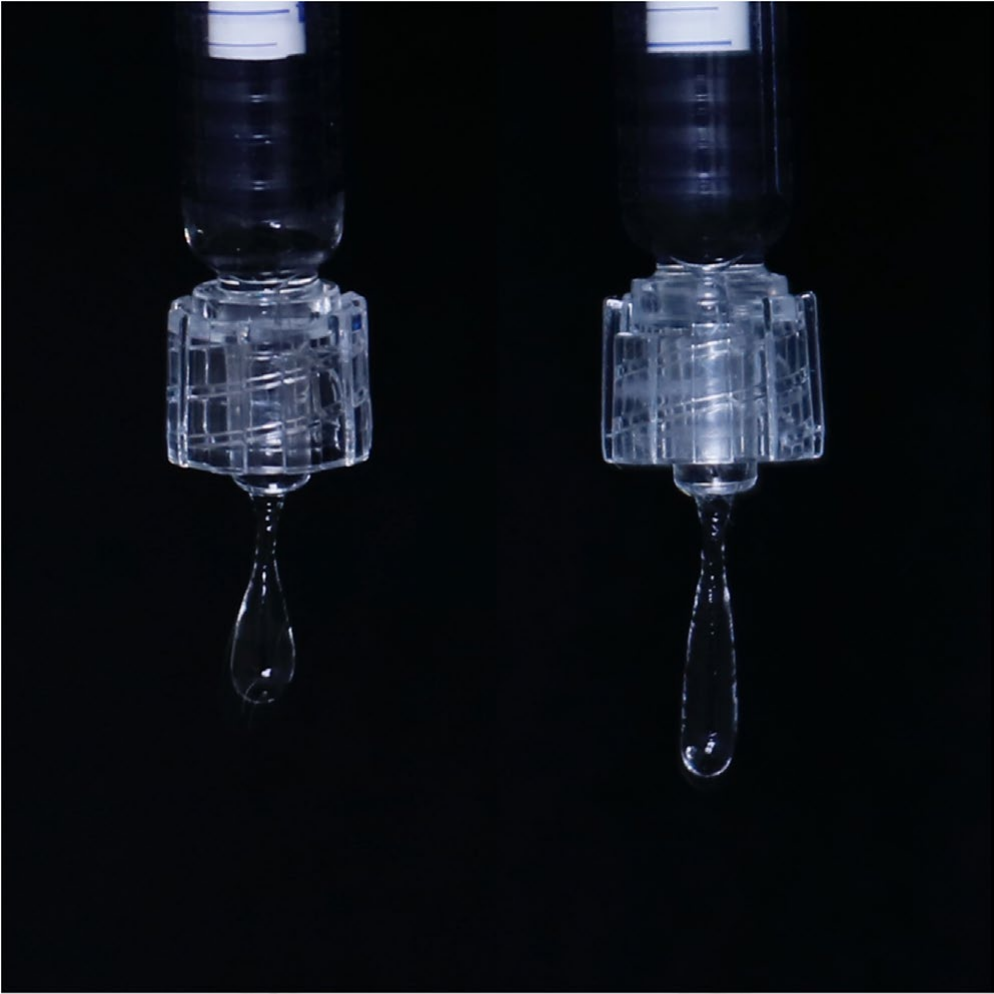
Drop weight comparison of two gel‐type HA products. The higher the cohesiveness, the longer the droplet and the greater the drop weight. Left: low‐cohesive HA (Aqualuna Feiran, for illustrative purposes only), suitable for lip injections; Right: high‐cohesive HA (Aqualuna Yaoran, for illustrative purposes only), suitable for deep midfacial injections.

Compared with granular fillers, gel fillers have markedly lower *G*′ and weaker vertical support, but significantly higher cohesivity. Thus, understanding cohesivity is critical. Cohesivity is defined as the capacity of a material not to dissociate, because of the affinity of its molecules for each other [8]. Highly cohesive gels aggregate in vivo, while less cohesive gels diffuse more readily. For treatments requiring localized, concentrated volumization, highly cohesive gels are preferred; when broader diffusion and uniform distribution are desired, less cohesive gels are more suitable. A simple clinical technique for estimating cohesivity is to express a droplet from the syringe: larger droplets indicate higher cohesivity, smaller droplets indicate lower (Figure 1).

Therefore, by distinguishing between gel and granular fillers, and further considering particle size for granular fillers or cohesivity for gels, clinicians can more efficiently and accurately match HA products to the five categories of indications. This classification of indications and the corresponding logic for hyaluronic acid filler selection are summarized based on the authors' clinical experience from Southern Chinese women. They have not been systematically or rigorously validated, nor are they widely recognized expert consensus or directly applicable clinical guidelines. Nevertheless, this framework has indeed helped the authors refine their indication assessment and filler selection process, which may lead to improved efficiency and patient satisfaction. We therefore carefully summarize our experience and present it as follows.

## 2 Indications for HA Fillers and Product Selection

### 2.1 Contouring Indications: Medium‐to‐Large Particle HA Fillers

Contouring indications refer to cases requiring robust structural support to enhance local bony prominence. Clinically, this primarily involves the supraorbital–nasal region (T‐zone) and the chin–mandibular line (V‐zone) [9, 10, 11, 12, 13, 14]. In East Asian populations, facial morphology tends to be relatively flat, with insufficient projection in the T‐zone and V‐zone. Moreover, the superficial soft tissues in these regions are denser and thicker, making it essential to establish strong and stable support to achieve bony contour definition.

**FIGURE 2:**
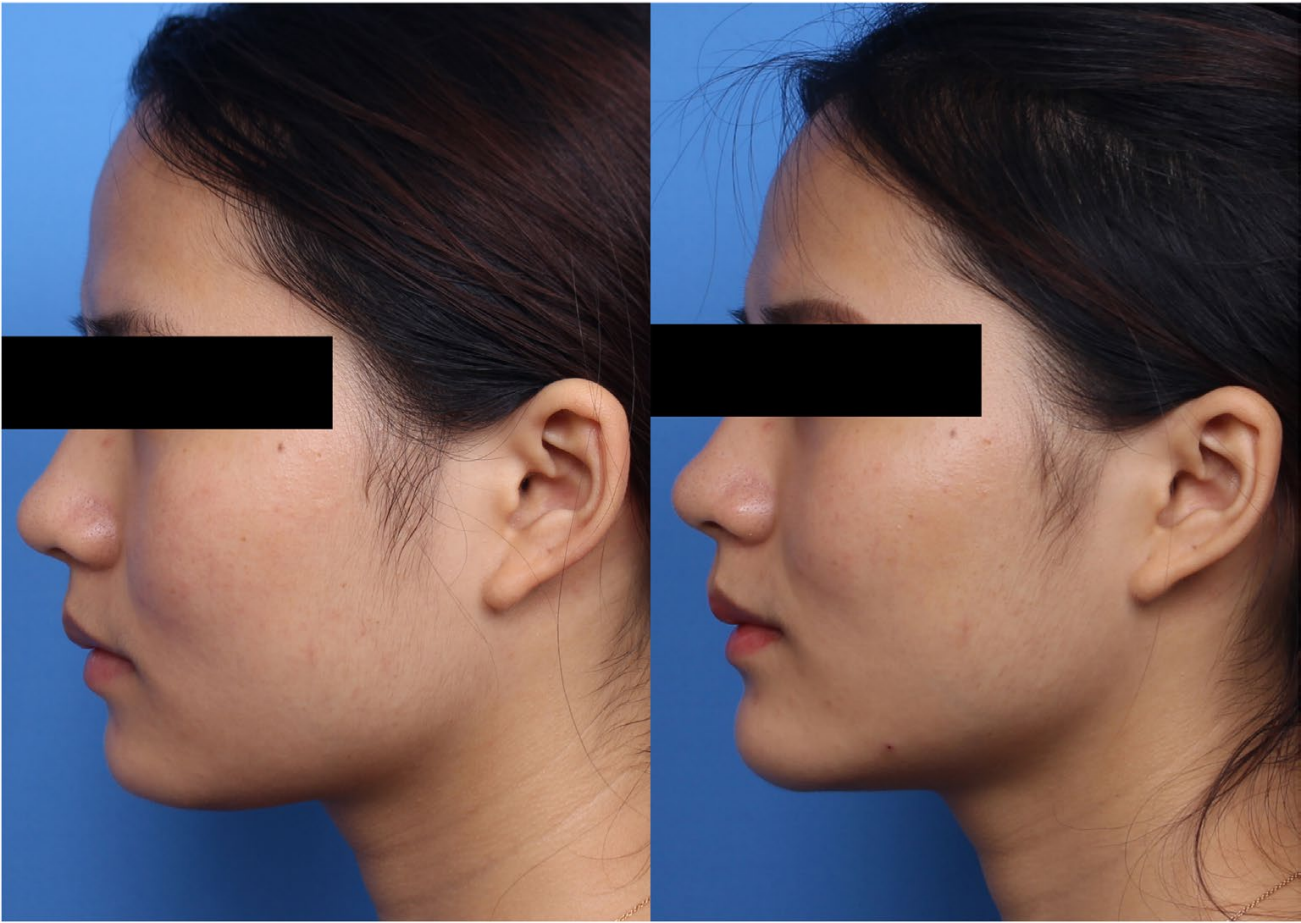
A female patient in her 20s treated with large‐particle HA filler (particle size exceeding 800 μm, *G*′ ~732 Pa; Aqualuna 5, 3 mL; for illustrative purposes only), achieving a well‐defined chin and mandibular contour with prominent projection and smooth curvature. The chin contour was harmonized with the mandibular line, elongating the lower face and improving overall facial proportions. Left: before injection; Right: immediately after injection, the cannula entry point at the mandibular line is visible.

Large‐particle HA fillers are characterized by a higher elastic modulus, or greater firmness. This property enables them to create contours with pronounced projection and sharp curvature—such as simulating the supraorbital ridge, nasal bones and cartilages in the T‐zone, and the mandibular border in the V‐zone (Figure 2). Their tendency to distribute vertically within tissues [4] helps maintain a narrow, well‐defined contour, which is especially suitable for these areas.

Product selection should consider both the mechanical demands of contouring and the thickness of overlying soft tissue. Sites requiring stronger mechanical support and deeper placement benefit from large‐particle fillers, whereas areas with lower demands or thinner soft tissues can be treated with medium‐particle fillers, which reduce the risk of product visibility or unnatural appearance. Although placing large‐particle fillers in superficial planes can efficiently generate contour projection and bony definition, such techniques require experienced injectors to balance strong support with natural aesthetics.

Given that both the T‐zone and V‐zone present as linear contours on the facial surface, linear injection techniques are most appropriate for contouring indications. In cases requiring enhanced projection, combining deep large‐particle fillers with superficial medium‐particle fillers can produce clearer and more refined bony contours.

### 2.2 Volumization Indications: Gel‐Type HA Fillers

In addition to contour enhancement of the T‐ and V‐zones, a second broad category of clinical demand involves restoring volume in areas of soft tissue depression. These volumization indications primarily include the temples, the superficial maxilla (mid‐cheek and nasolabial fold), lips, and pretarsal roll (“aegyo‐sal”) [15, 16, 17, 18, 19]. These sites provide favorable anatomical planes and spaces for HA filler placement: in the temporal region, fillers can be injected superficial to the deep temporal fascia; in the midface, both deep and superficial fat compartments are accessible; in the lips and pretarsal roll, injections can be placed in the superficial muscular plane beneath the mucosa or skin.

Unlike contouring indications, which emphasize sharp osseous contours, volumization indications focus on replicating the texture of fat or muscle. This explains the clinical preference for gel‐type HA fillers in such cases. Nonetheless, product selection and injection technique differ by site. For example, temporal augmentation requires smooth, broad contours; this can be facilitated by pre‐infiltration with local anesthetic or dilution of the gel filler, followed by fan‐shaped cannula placement to ensure even distribution [20]. For midface augmentation over the superficial maxilla, highly cohesive products are preferable, delivered with bolus injections via sharp needle to create light‐reflecting highlights. In contrast, lip and pretarsal roll augmentation require softer, less cohesive gels, as firmer products readily cause nodularity.

**FIGURE 3:**
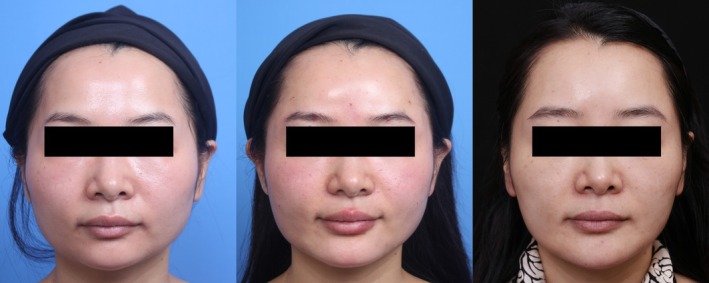
A female patient in her 30s underwent bilateral temporal augmentation with large‐particle HA (particle size exceeding 800 μm, *G*′ ~732 Pa; Aqualuna 5, supraperiosteal, 1 mL per side; for illustrative purposes only) and high‐cohesive gel‐type HA diluted with lidocaine at 1:0.3 (Aqualuna Natural, superficial to the deep temporal fascia, 1 mL each per side; for illustrative purposes only). Temporal volume was significantly restored with smooth, natural contour transitions at the temporofrontal and temporo‐orbital junctions. Left: before injection; Middle: immediately after injection; Right: 55 months after injection, the volume of temporal area remained.

For patients with pronounced hollowness and significant volume loss, particularly in the temples and midface, optimal outcomes are often achieved by combining deep and superficial injections. Deep placement on the supraperiosteal plane with large‐particle fillers (as in contouring indications) provides structural reinforcement, while superficial gel injections restore soft tissue fullness (Figure 3). In the temple, additional subcutaneous injection may further enhance volumization [15]. Lip and pretarsal roll augmentation can also be performed in two planes—intramuscular and submucosal or subdermal—to achieve greater fullness while preserving natural softness and avoiding induration.

### 2.3 Smoothening Indications: Medium‐to‐Small Particle or Low‐Cohesivity Gel HA Fillers

**FIGURE 4:**
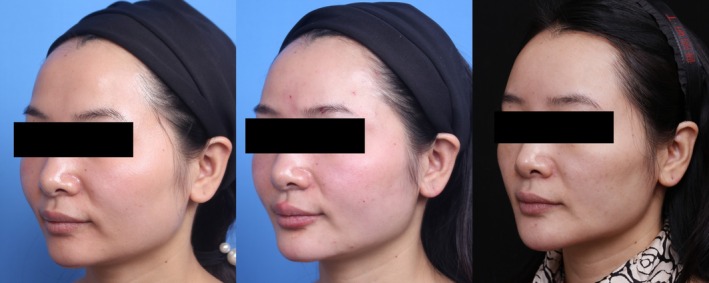
The same female patient in Figure 3 treated with medium‐particle HA (particle size ~550 μm, *G*′ ~579 Pa, Aqualuna 3, 2 mL; for illustrative purposes only), resulting in focal light reflection and a transition from a flat or mildly concave forehead to a slightly convex contour. Left: before injection; Middle: immediately after injection; Right: 55 months after injection, the appearance of forehead remained improved.

Unlike contouring or volumization indications, which generally require more pronounced changes, smoothing indications—such as forehead or subzygomatic hollowing—often call for only subtle adjustments. These sites have firm deep structures, namely the frontal bone and the parotid–masseteric fascia, while the overlying soft tissues are relatively thin. For this reason, medium‐to‐small particle fillers or soft, low‐cohesivity gels are recommended (Figure 4). Using large‐particle or highly cohesive fillers, or injecting excessive volumes, risks palpable nodularity [21, 22].

Injection planes also differ between the two regions. In the forehead, the galea aponeurotica is tightly adherent to the dermis, and numerous branches of the supratrochlear and supraorbital arteries run within the subcutaneous layer, increasing the risk of vascular embolism [23]. Deep subgaleal injection is therefore advisable. By contrast, in the subzygomatic hollow, filler can be injected directly into the subcutaneous layer.

### 2.4 Lifting Indications: Medium‐to‐Large Particle HA Fillers

Lifting indications refer to cases where filler‐induced structural support at injection points displaces adjacent or even distant fascial and ligamentous tissues, producing a visible lifting effect. Clinically, this primarily includes injections at ligament anchoring points, the posterior temporal region, and broader outer contour sites [15, 24, 25, 26, 27, 28].

Research on ligament‐based injections is relatively earlier. Anatomical and clinical studies have shown that injection beneath true retaining ligaments elevates the ligament and the soft tissues tethered to it, thereby producing a lifting effect [29]. Although this technique has demonstrated efficacy [24, 25, 26, 27, 30], its duration is often limited. To enhance both the magnitude and longevity of lifting, broader‐area injections with larger product volumes—such as posterior temporal injections—have been explored. Experts have proposed the concept of using ligament lines to delineate inner and outer facial contours [31], and introduced the surface–volume coefficient (SVC) as a quantitative index for assessing the lifting potential of HA fillers [32].

Current understanding of ligament and contour lifting [33, 34] suggests that comprehensive restoration of upper and lateral tissue volume, reinforced by fibrous connections between compartments, enables filler‐induced support to displace medial and inferior soft tissues upward and laterally, resulting in clinically visible lifting. To generate adequate support, medium‐to‐large particle fillers are recommended for ligament‐based and contour lifting injections. Unlike contouring indications, which typically employ continuous linear injection, lifting injections should avoid tissue separation and continuous linear deposition. Instead, they emphasize bead‐like bolus placement within individual fibrous compartments, preserving septal integrity and thereby enhancing lifting strength [27].

**FIGURE 5:**
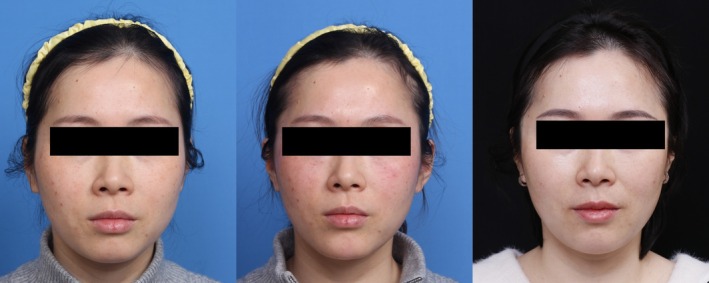
A female patient in her 30s received multi‐site injection with medium‐particle HA filler (particle size 350~500 μm, *G*′ ~450 Pa, Fumeideng; for illustrative purposes only), totaling 23 mL: Posterior temporal (4 + 4 mL, deep and superficial), preauricular (2 mL), postauricular (6 mL), and mandibular angle (2 mL) for lifting; supraorbital ridge (1 mL) for contouring; anterior temporal region (3 mL), and infraorbital maxilla (1 mL) for volumization. Left: before injection; Middle: immediately after injection; Right: 2 years after injection, the facial curve improved and the lifting effect remained.

Outer contour lifting generally requires larger filler volumes, often 10 mL or more in a single session. When product selection and injection technique are appropriate, the lifting effect can be striking (Figure 5), with visible improvement of nasolabial and labiomental folds. Nevertheless, clinical experience with ligament and contour lifting remains relatively limited; the underlying mechanisms of action have not yet been thoroughly elucidated, and the long‐term efficacy awaits further investigation. In addition, the relatively large injection dosage required may increase the risk of vascular injury or induce compressive ischemia secondary to elevated local tissue tension, and complications such as alopecia have been reported [35]. Thus, wider clinical adoption of lifting indications requires further in‐depth and long‐term study, and high‐volume injection shouldn't be taken as a routine recommendation. Its potential risks are often overshadowed by more prominent outcomes and greater benefits, making them easily overlooked. Therefore, caution should be exercised when considering high‐dose hyaluronic acid injection, and extreme care and close observation are required during actual administration.

### 2.5 Revitalization Indications: Ultra‐Small Particle, Non‐Crosslinked, or Diluted Gel‐Type HA

Unlike conventional HA filler techniques, ultra‐small particle and non‐crosslinked HA formulations have recently emerged for dermal and superficial subcutanous injection [36, 37, 38]. Ultra‐small particle products have a smaller particle size than small‐particle fillers, while non‐crosslinked HA undergoes more rapid metabolic degradation, both making them suitable for superficial application. Physicians accustomed to gel‐type fillers have also adopted diluted gel‐type HA for superficial subcutaneous injection [39, 40].

**FIGURE 6:**
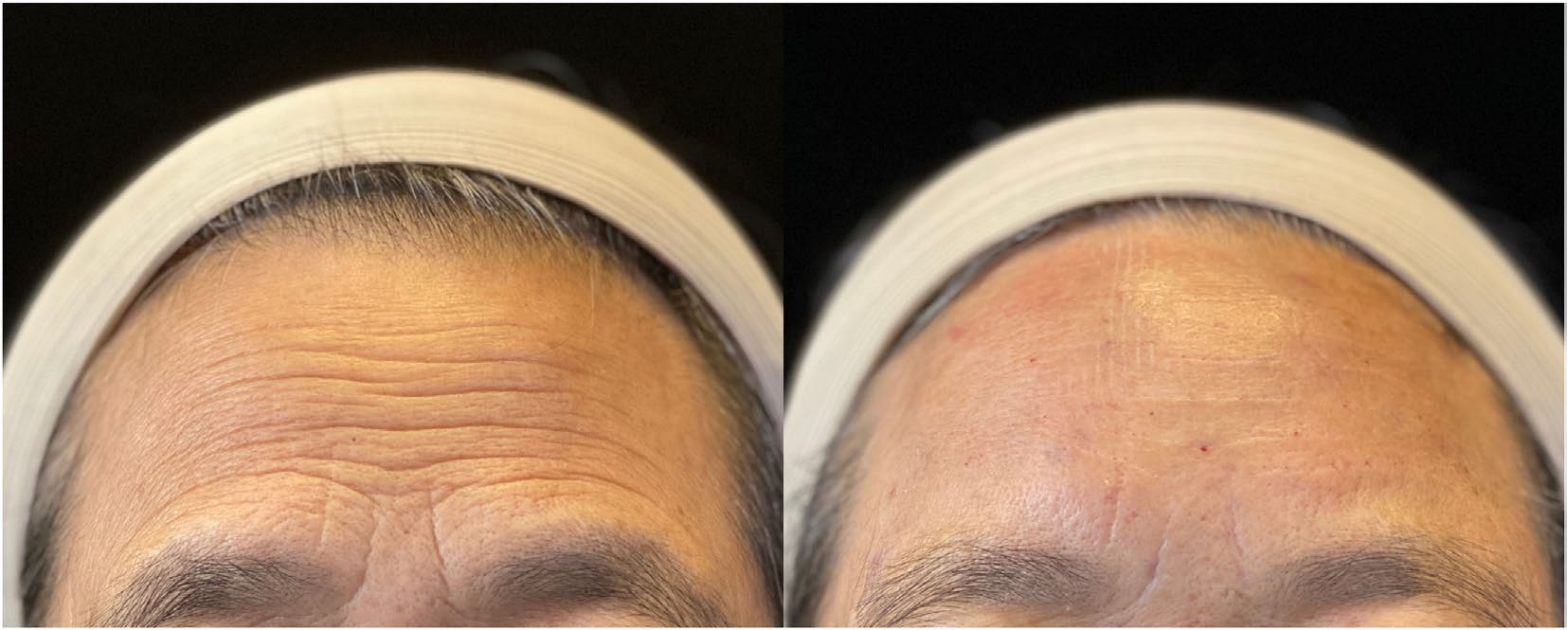
A female patient in her 70s was treated for forehead and glabellar rhytids with intradermal injection of ultra‐small‐particle HA filler (particle size ~200 μm, *G*′ ~230 Pa, Aqualuna Aqua, 1.5 mL; for illustrative purposes only) combined with botulinum toxin (Hengli, 20 U; for illustrative purposes only). Left: before injection; Right: immediately after injection.

Dilution of HA fillers significantly reduces their support and cohesiveness, promoting spread and diffusion in vivo. Based on the author's clinical experience, not only gel‐type but also particle‐type fillers can be diluted to yield HA fillers better suited for superficial use. Such dermal or immediately subdermal applications are primarily employed for targeted correction of fine lines such as forehead wrinkles, glabellar lines, nasolabial folds, cheek lines, or perioral lines (Figure 6), and have also been widely applied in mesotherapy for dermal rejuvenation. In fact, the earliest HA fillers were introduced as dermal fillers, emphasizing their use within the dermis. However, if the filler has a larger particle size (particle‐type) or higher concentration with stronger cohesiveness (gel‐type), nodularity may occur after injection [41]. In contrast, ultra‐small particle, non‐crosslinked, or diluted gel‐type HA products better adapt to the thickness and texture of the dermis, representing the return of HA as a “dermal filler.”

For forehead wrinkle injection, subcutaneous placement is not recommended for safety reasons [23]; intradermal or immediate subdermal injection is safer. In contrast, correction of forehead contour irregularities under the third category of smoothening indications should employ subgaleal placement. Even when small‐particle or diluted gel‐type fillers are used, meticulous and even injection technique is required. Excessive injection may still result in nodularity, which often resolves poorly on its own. Thus, small‐particle or diluted gel‐type fillers are best suited for injection into the immediate subdermal layer. Non‐crosslinked HA is less prone to nodularity and is therefore more suitable for intradermal use.

It is also noteworthy that HA fillers, through biomechanical effects, can stimulate fibroblasts to secrete collagen [42, 43]. This underlies the skin revitalization outcomes achieved with superficial application of HA.

## 3 Discussion

**FIGURE 7:**
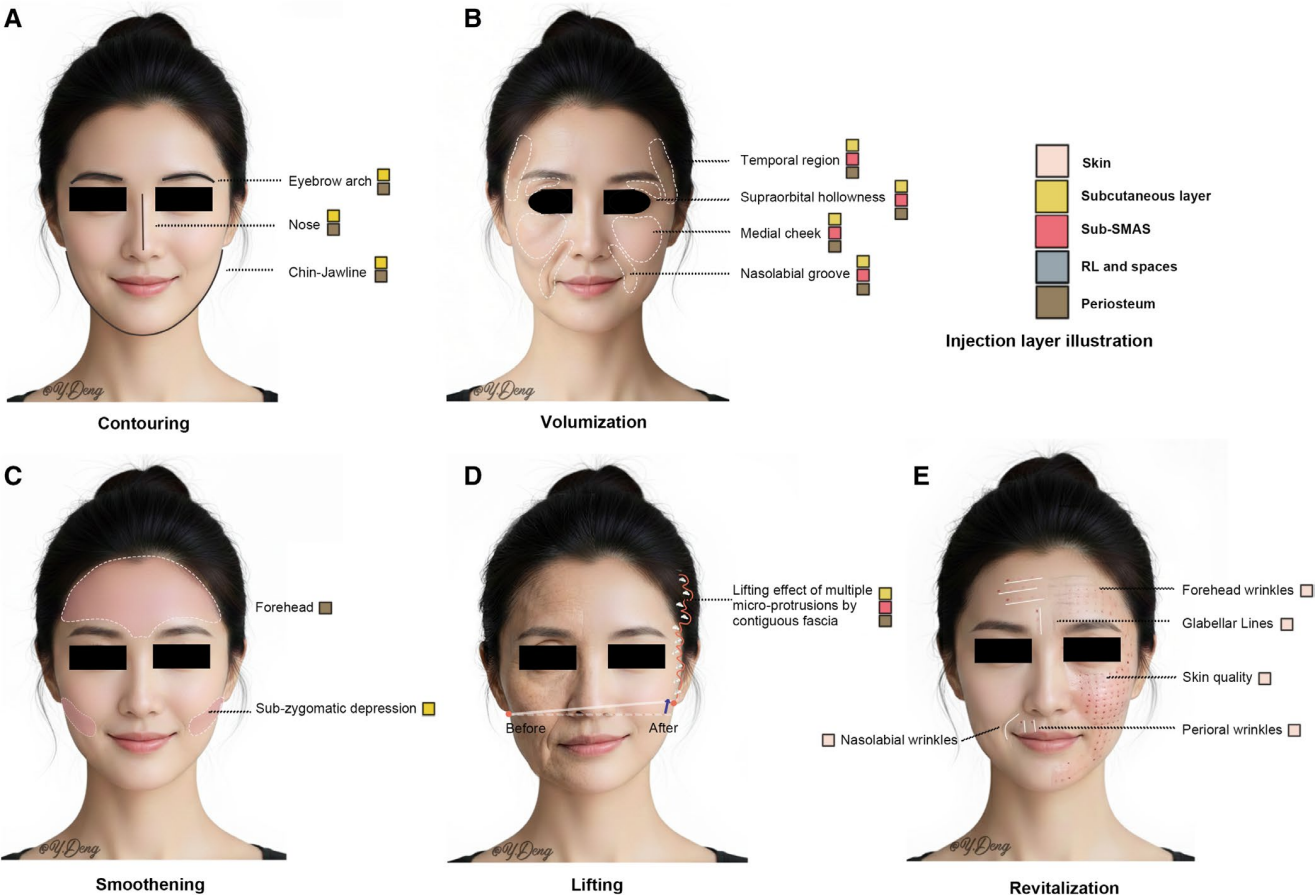
Schematic diagram of five indications for facial hyaluronic acid fillers. (A) Contouring indications, mainly covering the brow‐nasal region (T‐zone) and the chin‐mandibular border (V‐zone), for enhancing the prominence of linear bony contours. (B) Volumization indications, mainly involving the temporal region and the superficial soft tissue layer of the maxilla (mid‐cheek area, nasolabial folds) and other sites, focusing on volume replenishment for soft tissue depressions, which can be implemented with layered injection. (C) Smoothening indications, where only a small amount of hyaluronic acid filler needs to be injected into the subgaleal or subcutaneous layer for subtle improvement when addressing frontal depressions and subzygomatic depressions. (D) Lifting indications, mainly including various ligament points, the posterior temporal region and broader external contour injection areas, characterized by a series of discrete small bolus injections creating multiple micro‐protrusions to pull the fascia upward, thereby achieving a lifting effect for the area below the injection site. (E) Rejuvenation indications, mainly targeting the improvement of frontal lines, glabellar lines, nasolabial folds, cheek lines, perioral lines, and other facial wrinkles, or intradermal injection for mesotherapy to improve skin quality.

With deeper insights into HA filler materials, facial anatomy and aging processes, physicians can flexibly use various products for facial contouring, volumizing, smoothing, lifting and skin rejuvenation (Table 1). Previous authors have proposed a similar framework, classifying HA injection into five indications—contouring, lifting, volumization, smoothening, and skin boosting [44]—with corresponding filler selection strategies. The present classification seems to share substantial overlap with Kapoor et al.'s system. However, our approach differs in two significant aspects. First, this article defines five indications based on the desired therapeutic effects rather than solely relying on injection layers. Consequently, specific injection sites are provided for each of these five indications, enabling more targeted alignment with the cosmetic concerns that patients present to the clinic. Second, a stratified analysis of the causes of aging facilitates our understanding of its intrinsic nature; however, aging progresses synchronously across multiple levels. Therefore, we advocate for combined multi‐level therapy. As mentioned earlier, for indications related to contouring, volumization, and lifting, we suggest multi‐layer treatment when necessary to achieve optimal outcomes. Such combined multi‐layer application can enhance the efficacy of HA filling, which is particularly essential for East Asian individuals with thicker soft tissues. Rejuvenation indications can be adopted for most cases requiring skin quality improvement. In contrast, although smoothening indications do not involve multi‐level combination injections, they can be applied in a coordinated manner across different facial regions. For these five types of indications, we have also summarized their respective characteristics into a schematic diagram for ease of understanding (Figure 7). Therefore, comparing with Kapoor et al.'s classification, although the five indications are nearly identical in terms of their nomenclature, there are distinct differences in their therapeutic objectives, injection principles, specific injection techniques, and material applications. Moreover, a true understanding of the five indications extends beyond recognizing their individual features and appropriate filler choices. It is equally important to emphasize combined applications, whether simultaneous or sequential, targeting multiple indications to deliver comprehensive improvements and higher patient satisfaction. Typically, when addressing multiple indications, the priority sequence is: lifting → contouring → volumization → smoothening → revitalization. This order better conforms to biomechanical principles and aesthetic logic.

**TABLE 1:** Five facial indications, expected effects, HA filler selection and key points.

Indication	Specific areas	Expected effect	Product selection	Key points
Contouring	Eyebrow arch—Nose (T‐zone), Chin—Jawline (V‐zone)	Strong bony contour	Medium to large particle size granular HA	Choose large particle size for deep layers and medium particle size for superficial layers
Volumization	Temporal, Supraorbital hollowness, Medial cheek, Nasolabial groove, Lips, Pretarsal roll	Plump Volumetric filling	Gel‐type/combined with large particle size granular HA	Choose high cohesive HA for projected areas and low cohesive HA for flat areas
Smoothening	Forehead and Sub‐zygomatic depression	Smooth transition	Small to medium particle size granular or low‐cohesive gel‐type HA	Small amount and uniform injection
Lifting	Various ligament points, Posterior temporal region, and broader superior lateral regions	Lifting from inner‐lower to outer‐upper direction	Medium to large particle size granular HA	Point bolus injection, avoid tissue separation, and reduce damage to fibrous septa
Revitalization	Forehead wrinkles, Glabellar lines, Nasolabial folds, Cheek lines, Perioral wrinkles, Full‐face meso‐therapy	Superficial micro‐filling, Dermal revitalization	Ultra‐small particle size granular, non‐cross‐linked, or diluted gel‐type HA	Small amount per point and uniform injection

Corresponding to these five types of indications is the selection of hyaluronic acid products. What is challenging is that aesthetic physicians are now faced with a growing number of hyaluronic acid brands. Taking the Chinese region where the authors are based as an example, more than 60 brands and 100 categories of hyaluronic acid products are legally available for purchase. What is even more challenging is that different brands often claim to be the optimal choice for a specific indication. Although many experts have conducted extensive research on the physicochemical properties and rheological parameters of hyaluronic acid, there remain numerous obscure and even contradictory findings. Guiding the selection of hyaluronic acid products based on laboratory data has not yet become a common practice. For this reason, the authors of this paper have been exploring a scientific yet concise strategy for hyaluronic acid product selection in clinical practice. Regarding the particle size of hyaluronic acid products, it can usually be clearly indicated in the product instructions and can also be perceived through manual palpation and kneading. In addition, droplet size is another simple yet scientific index for evaluating hyaluronic acid properties, which can be easily assessed right in the clinic. In other words, physicians can obtain relatively clear results for these two parameters by themselves, which are more intuitive and practically meaningful compared with the rheological data measured in specialized materials science laboratories. In our clinical practice and communications with numerous physicians, we have found that this summary has aroused widespread interest and attention. Therefore, we are willing to share this concise classification of facial injection indications and the corresponding hyaluronic acid filler selection strategy for reference. At present, the development of minimally invasive injection aesthetics in East Asia is rapidly evolving, yet the related injection concepts and clinical experience remain relatively lagging, and there is a lack of unified standards and systematic frameworks. Prior to our work, many experts have carried out similar research and summarization [43, 44, 45, 46]. These experiences and summaries have provided positive guidance for us to establish contouring standards and product selection logic. Hence, we put forward our own clinical experience, hoping to help physicians who are confused about indication selection and product choice, and we also look forward to further discussions and corrections from our peers in the field.

It is important to emphasize that this paper is merely a summary of clinicians' personal experience applied in clinical practice. Although it incorporating research findings from anatomy, materials science, and clinical therapeutic practice, it still has obvious limitations. First, the manuscript is essentially experience‐based expert synthesis without robust statistical efficacy evaluation or controlled comparative data. Second, this experience is mainly based on female patients in Southern China, and thus may not be applicable to patients of other regions, ethnicities, or genders. For instance, the subzygomatic depression usually requires correction in East Asians, whereas it often needs to be preserved in Caucasians. When applying the experience described in this article to other patient populations, further discussion and modification are still needed based on patient needs and physician practice patterns.

Although this article classifies facial aesthetic injections, it does not imply that these five categories represent completely distinct clinical indications. In fact, filler injection serves as the foundation for the clinical therapeutic effects of all facial fillers; it can often simultaneously achieve multiple outcomes such as volumization, contouring, lifting, and skin revitalization. The core focus of this article lies in the details and techniques that determine whether the post‐injection result emphasizes bony contours, soft tissue fullness, or lifting and firming effects. We intend to help aesthetic physicians reach targeted treatment goals in a concise way, instead of enforcing rigid classification of the five indications. Similar to most studies, our filler efficacy evaluation and indication grouping are summarized from clinical experience, not unified standard protocols.

Finally, it should be noted that while this article emphasizes product selection based on indication classification, the properties of HA fillers cannot be fully captured by simple indices such as particle versus gel form, particle size, or cohesiveness. The proposed indication‐based product‐matching strategy is intended merely as a practical reference. Proper material selection alone cannot guarantee ideal results. Anatomical knowledge, personalized evaluation, and skilled injection techniques are equally vital clinically. Nonetheless, under otherwise equal conditions, scientifically and rationally selecting the filler material contributes to superior results and greater patient trust. The science of injectable materials is still evolving and advancing rapidly, with novel concepts and approaches constantly being proposed [47, 48, 49, 50, 51]. Therefore, clinicians need to keep abreast of relevant progress and update their knowledge on an ongoing basis to ensure appropriate product selection and application, thereby optimizing treatment outcomes.

## 4 Conclusion

This article, addressing the demands of facial contouring and rejuvenation, categorizes HA injection into five clinical indications: contouring, volumization, smoothening, lifting, and revitalization. It distinguishes the characteristics of particle‐type (large, medium, small, and ultra‐small particle sizes) and gel‐type (high versus low cohesiveness) fillers, and proposes the core strategy of “indication‐based product matching”‐for example, medium‐ to large‐particle fillers for contouring, gel‐type fillers for volumization, etc. It further introduces the priority order for combined treatments—lifting → contouring → volumization → smoothening → revitalization—while underscoring that anatomical familiarity and injection technique are also important in determining clinical outcomes. Together, these principles provide a concise and practical reference for clinical HA filler application.

## Author Contributions

Conceptualization and design: Donghui Wu; original draft preparation: Hao Feng, Ting Wang, Donghui Wu; writing – editing: Donghui Wu, Liangsen Zhao; review: Hao Feng, Caijin Xiang, Xiang Liu, Haihua Li, Yong Liao; supervision: Yong Liao, Donghui Wu; all authors approved the final manuscript and agreed to submission.

## Funding

The authors have nothing to report.

## Ethics Statement

This study involves case treatments that have obtained approval from the involved medical aesthetic clinics. This work does not involve clinical trials or animal experiments, and all relevant procedures comply with the ethical guidelines for medical aesthetic practice.

## Consent

Written informed consent was obtained from all patients for the performance of hyaluronic acid filler treatment and the publication of all treatment details, follow‐up data, and clinical images included in this article.

## Conflicts of Interest

Caijin Xiang, Xiang Liu, Haihua Li, and Donghui Wu are consultants for Bloomage Biotechnology Corporation Limited (Beijing, China). The other authors declare no conflicts of interest.

## Data Availability

The data supporting this study's findings are available from the corresponding author (Donghui Wu) upon reasonable request, and are not publicly available due to patient clinical information privacy and institutional data management policies.
